# On a problem of Pillai with Fibonacci numbers and powers of 3

**DOI:** 10.1007/s40590-019-00263-1

**Published:** 2019-09-17

**Authors:** Mahadi Ddamulira

**Affiliations:** Institute of Analysis and Number Theory, Graz University of Technology, Kopernikusgasse 24/II, 8010 Graz, Austria

**Keywords:** Fibonacci numbers, Linear forms in logarithms, Baker’s method, 11B39, 11J86

## Abstract

Consider the sequence $$ \{{F}_{n}\}_{n \ge 0} $$ of Fibonacci numbers defined by $${F}_0=0$$, $${F}_1 =1$$, and $${F}_{{n}+2}= {F}_{{n}+1}+ {F}_{n} $$ for all $$ n\ge 0 $$. In this paper, we find all integers *c* having at least two representations as a difference between a Fibonacci number and a power of 3.

## Introduction

We consider the sequence $\{F_{n}\}_{n\ge 0}$ of Fibonacci numbers defined by

$$\begin{aligned} F_0=0, ~F_1= 1, \text { and } F_{n+2}= F_{n+1}+F_n \, \text { for all } \, n\ge 0. \end{aligned}$$

The first few terms of the Fibonacci sequence are

$$\begin{aligned} \{F_{n}\}_{n\ge 0} = 0, 1, 1, 2, 3, 5, 8, 13, 21, 34, 55, 89, 144, 233, \ldots . \end{aligned}$$

In this paper, we are interested in studying the Diophantine equation

1 $$\begin{aligned} F_n - 3^m = c \end{aligned}$$

for a fixed integer *c* and variable integers *n* and *m*. In particular, we are interested in finding those integers *c* admitting at least two representations as a difference between a Fibonacci number and a power of 3. This equation is a variant of the Pillai equation

2 $$\begin{aligned} a^{x}-b^{y}=c \end{aligned}$$

where *x*, *y* are nonnegative integers and *a*,  *b*,  *c* are fixed positive integers.

In 1936 and again in 1937, Pillai (see [16, 17]) conjectured that for any given integer $c\ge 1$, the number of positive integer solutions (*a*, *b*, *x*, *y*) , with $x\ge 2$ and $y\ge 2$ to the Eq. (2) is finite. This conjecture is still open for all $c\ne 1$. The case $c=1$ is Catalan’s conjecture which was proved by Mihăilescu (see [15]). Pillai’s work was an extension of the work of Herschfeld (see [12, 13]), who had already studied a particular case of the problem with $(a,b)=(2,3)$. Since then, different variants of the Pillai equation have been studied. Some recent results for the different variants of the Pillai problem involving Fibonacci numbers, Tribonacci numbers, Pell numbers and the *k*-generalized Fibonacci numbers with powers of 2 have been intensively studied in [3–7, 10, 11].

## Main result

The main aim of this paper is to prove the following result. Since $F_1=F_2=1$, we discard the situation when $n=1$ and just count the solutions for $n=2$.

### Theorem 1

The only integers *c* having at least two representations of the form $F_{n}-3^{m}$ are $c\in \{ -26, -6, -1, 0, 2, 4, 7, 12\}$. Furthermore, all the representations of the above integers as $F_{n}-3^{m}$ with integers $n\ge 2$ and $m\ge 0$ are given by

3 $$\begin{aligned} -26= & {} F_{10} - 3^4 = F_2-3^3;\nonumber \\ -6= & {} F_8-3^3 = F_4 - 3^2;\nonumber \\ -1= & {} F_6-3^2 = F_3-3^1\nonumber \\ 0= & {} F_4-3^1 = F_2-3^0;\nonumber \\ 2= & {} F_5-3^1 = F_4-3^0;\nonumber \\ 4= & {} F_7-3^2 = F_5-3^0;\nonumber \\ 7= & {} F_9-3^3=F_6-3^0;\nonumber \\ 12= & {} F_8-3^2=F_7-3^0. \end{aligned}$$

## Auxiliary results

To prove our main result Theorem 1, we need to use several times a Baker-type lower bound for a nonzero linear form in logarithms of algebraic numbers. There are many such in the literature like that of Baker and Wüstholz from [2]. We use the one of Matveev from [14]. Matveev [14] proved the following theorem, which is one of our main tools in this paper.

Let $\gamma$ be an algebraic number of degree *d* with minimal primitive polynomial over the integers

$$\begin{aligned} a_{0}x^{d}+ a_{1}x^{d-1}+\cdots +a_{d} = a_{0}\prod _{i=1}^{d}(x-\gamma ^{(i)}), \end{aligned}$$

where the leading coefficient $a_{0}$ is positive and the $\eta ^{(i)}$’s are the conjugates of $\gamma$. Then, the *logarithmic height* of $\gamma$ is given by

$$\begin{aligned} h(\gamma ) := \dfrac{1}{d}\left( \log a_{0} + \sum _{i=1}^{d}\log \left( \max \{|\gamma ^{(i)}|, 1\}\right) \right) . \end{aligned}$$

In particular, if $\gamma = p/q$ is a rational number with $\gcd (p,q) = 1$ and $q>0$, then $h(\gamma ) = \log \max \{|p|, q\}$. The following are some of the properties of the logarithmic height function $h(\cdot )$, which will be used in the next sections of this paper without reference:

4 $$\begin{aligned} h(\eta \pm \gamma )\le & {} h(\eta ) +h(\gamma ) +\log 2,\nonumber \\ h(\eta \gamma ^{\pm 1})\le & {} h(\eta ) + h(\gamma ),\nonumber \\ h(\eta ^{s})= & {} |s|h(\eta ) ~~~~~~ (s\in {\mathbb {Z}}). \end{aligned}$$

### Theorem 2

(Matveev) Let $\gamma _1,\ldots ,\gamma _t$ be positive real algebraic numbers in a real algebraic number field ${\mathbb {K}}$ of degree *D*, $b_1,\ldots ,b_t$ be nonzero integers, and assume that

5 $$\begin{aligned} \Lambda :=\gamma _1^{b_1}\cdots \gamma _t^{b_t} - 1, \end{aligned}$$

is nonzero. Then

$$\begin{aligned} \log |\Lambda | > -1.4\times 30^{t+3}\times t^{4.5}\times D^{2}(1+\log D)(1+\log B)A_1\cdots A_t, \end{aligned}$$

where

$$\begin{aligned} B\ge \max \{|b_1|, \ldots , |b_t|\}, \end{aligned}$$

and

$$\begin{aligned}A _i \ge \max \{Dh(\gamma _i), |\log \gamma _i|, 0.16\},\quad {\text {for all}}\quad i=1,\ldots ,t. \end{aligned}$$

During the calculations, we get upper bounds on our variables which are too large; thus, we need to reduce them. To do so, we use some results from the theory of continued fractions.

For the treatment of linear forms homogeneous in two integer variables, we use the well-known classical result in the theory of Diophantine approximation.

### Lemma 1

Let $\tau$ be an irrational number, $\frac{p_0}{q_0}, \frac{p_1}{q_1}, \frac{p_2}{q_2}, \ldots$ be all the convergents of the continued fraction of $\tau$ and *M* be a positive integer. Let *N* be a nonnegative integer such that $q_N> M$. Then, putting $a(M):=\max \{a_{i}: i=0, 1, 2, \ldots , N\}$, the inequality

$$\begin{aligned} \left| \tau - \dfrac{r}{s}\right| > \dfrac{1}{(a(M)+2)s^{2}}, \end{aligned}$$

holds for all pairs (*r*, *s*) of positive integers with $0<s<M$.

For a nonhomogeneous linear form in two integer variables, we use a slight variation of a result due to Dujella and Pethő (see [8], Lemma 5a), which is itself a generalization of the result of Baker and Davenport [1]. For a real number *X*, we write $||X||:= \min \{|X-n|: n\in {\mathbb {Z}}\}$ for the distance from *X* to the nearest integer.

### Lemma 2

Let *M* be a positive integer, $\frac{p}{q}$ be a convergent of the continued fraction of the irrational number $\tau$ such that $q>6M$, and $A,B,\mu$ be some real numbers with $A>0$ and $B>1$. Let further $\varepsilon : = ||\mu q||-M||\tau q||$. If $\varepsilon > 0$, then there is no solution to the inequality

$$\begin{aligned} 0<|u\tau -v+\mu |<AB^{-w}, \end{aligned}$$

in positive integers *u*, *v*, and *w* with

$$\begin{aligned} u\le M \quad {\text {and}}\quad w\ge \dfrac{\log (Aq/\varepsilon )}{\log B}. \end{aligned}$$

Finally, the following lemma is also useful. It is Lemma 7 in [9].

### Lemma 3

(Gúzman, Luca) If $m\ge 1$, $T>(4m^2)^m$, and $T>x/(\log x)^m$, then

$$\begin{aligned} x<2^mT(\log T)^m. \end{aligned}$$

## Proof of Theorem 1

Assume that there exist nonnegative integers $n, m, n_1, m_1$ with $\min \{n, n_1\} \ge 2$ and $\min \{m, m_1\} \ge 0$ such that $(n,m)\ne (n_{1}, m_{1})$, and

$$\begin{aligned} F_n -3^m = F_{n_{1}}-3^{m_1}. \end{aligned}$$

Without loss of generality, we can assume that $m\ge m_1$. If $m=m_1$, then $F_n =F_{n_1}$, so $(n,m)= (n_{1}, m_{1})$, which gives a contradiction to our assumption. Thus, $m>m_1$. Since

6 $$\begin{aligned} F_n - F_{n_1} = 3^m -3^{m_1}, \end{aligned}$$

and the right-hand side is positive, we get that the left-hand side is also positive and so $n>n_{1}$.

Using the Binet formula

7 $$\begin{aligned} F_k = \dfrac{\alpha ^{k}- \beta ^k}{\sqrt{5}} \text { for all } k\ge 0, \end{aligned}$$

where $(\alpha ,\beta ):=\left( \frac{1+\sqrt{5}}{2}, \frac{1-\sqrt{5}}{2}\right)$ are the roots of the equation $x^2-x-1 =0$, which is the characteristic equation of the Fibonacci sequence. One can easily prove by induction that

8 $$\begin{aligned} \alpha ^{k-2}\le F_{n}\le \alpha ^{k-1} \text { for all } k\ge 1. \end{aligned}$$

Using the Eq. (6), we get

9 $$\begin{aligned}&\alpha ^{n-4}\le F_{n-2}\le F_n-F_{n_1}=3^m-3^{m_1} <3^m, \end{aligned}$$

10 $$\begin{aligned}&\alpha ^{n-1}\ge F_{n}\ge F_n-F_{n_1}=3^m-3^{m_1} \ge 3^{m-1}, \end{aligned}$$

from which we get that

11 $$\begin{aligned} 1+\left( \dfrac{\log 3}{\log \alpha }\right) (m-1)< n< \left( \dfrac{\log 3}{\log \alpha }\right) m+4. \end{aligned}$$

If $n\le 300$, then $m\le 127$. We ran a *Mathematica* program for $2\le n_{1}<n\le 300$ and $0\le m_1 < m\le 127$ and found only the solutions from the list (3). From now, we assume that $n> 300$ and from (11) we have that $m>127$. Therefore, to solve the Diophatine Eq. (1), it suffices to find an upper bound for *n*.

### Bounding *n*

By substituting the Binet formula (7) in the Diophantine Eq. (1), we get

$$\begin{aligned} \left| \dfrac{\alpha ^{n}}{\sqrt{5}}-3^{m}\right|= & {} \left| \dfrac{\beta ^{n}}{\sqrt{5}}+\dfrac{\alpha ^{n_1}-\beta ^{n_1}}{\sqrt{5}}-3^{m_1}\right| ~\le ~ \dfrac{\alpha ^{n_1}+2}{\sqrt{5}}+3^{m_1}\\\le & {} \dfrac{2\alpha ^{n_1}}{\sqrt{5}}+3^{m_1}~<~3\max \{\alpha ^{n_1}, 3^{m_1}\}. \end{aligned}$$

Multiplying through by $3^{-m}$, using the relation (9) and using the fact that $\alpha < 3$, we get

12 $$\begin{aligned} |(\sqrt{5})^{-1}\alpha ^{n}3^{-m} -1|< 3\max \left\{ \dfrac{\alpha ^{n_1}}{3^{m}}, 3^{m_{1}-m}\right\} <\max \{\alpha ^{n_1-n+7}, 3^{m_1-m+1}\}. \end{aligned}$$

For the left-hand side, we apply the result of Matveev, Theorem 2 with the following data:

$$\begin{aligned} t=3, ~~ \gamma _1=\sqrt{5}, ~~\gamma _2 = \alpha , ~~\gamma _3 = 3, ~~b_1=-1, ~~b_2 = n, ~~b_3=-m. \end{aligned}$$

Throughout we work with the field ${\mathbb {K}}:={\mathbb {Q}}(\sqrt{5})$ with $D=2$. Since $\max \{1,n,m\}\le 2n$, we take $B:=2n$. Furthermore, we take $A_1:=2h(\gamma _1) = \log 5$, $A_2:=2h(\gamma _2) = \log \alpha$, $A_3:=2h(\gamma _1) = 2\log 3$. We put

$$\begin{aligned} \Lambda = (\sqrt{5})^{-1}\alpha ^{n}3^{-m} -1. \end{aligned}$$

First we check that $\Lambda \ne 0$, if it were, then $\alpha ^{2n}\in {\mathbb {Q}}$, a contradiction. Thus, $\Lambda \ne 0$. Then, by Matveev’s theorem, the left-hand side of (12) is bounded as

$$\begin{aligned} \log |\Lambda |> -1.4\cdot 30^6\cdot 3^{4.5}\cdot 2^2(1+\log 2)(1+\log 2n)(\log 5)(\log \alpha )(2\log 3). \end{aligned}$$

By comparing with (12), we get

$$\begin{aligned} \min \{(n-n_1-7)\log \alpha , (m-m_1-1)\log 3\}<1.66\times 10^{12}(1+\log 2n), \end{aligned}$$

which gives

$$\begin{aligned} \min \{(n-n_1)\log \alpha , (m-m_1)\log 3\}<1.67\times 10^{12}(1+\log 2n). \end{aligned}$$

Now, we split the argument into two cases.

**Case 1.**$$\min \{(n-n_1)\log \alpha , (m-m_1)\log 3\} = (n-n_1)\log \alpha $$.

In this case, we rewrite (6) as

$$\begin{aligned} \left| \left( \dfrac{\alpha ^{n}-\alpha ^{n_1}}{\sqrt{5}}\right) -3^{m}\right| =\left| \left( \dfrac{\beta ^{n}-\beta ^{n_1}}{\sqrt{5}}\right) -3^{m_1}\right| ~<~ 1+3^{m_1}~\le ~3^{m_1+1}, \end{aligned}$$

which implies

13 $$\begin{aligned} \left| \left( \dfrac{\alpha ^{n-n_1}-1}{\sqrt{5}}\right) \alpha ^{n_1}3^{-m}-1\right|< & {} 3^{m_1-m+1}. \end{aligned}$$

We put

$$\begin{aligned} \Lambda _1 = \left( \dfrac{\alpha ^{n-n_1}-1}{\sqrt{5}}\right) \alpha ^{n_1}3^{-m}-1. \end{aligned}$$

To see that $\Lambda _1 \ne 0$, for if $\Lambda _1 =0$, then

$$\begin{aligned} \alpha ^{n}-\alpha ^{n_1}= \sqrt{5}\cdot 3^{m}. \end{aligned}$$

By conjugating the above relation in ${\mathbb {K}}$, we get that

$$\begin{aligned} \beta ^{n}-\beta ^{n_1}= -\sqrt{5}\cdot 3^{m}. \end{aligned}$$

The absolute value of the left-hand side is at most $|\beta ^{n}-\beta ^{n_1}|\le |\beta |^{n}+|\beta |^{n_1}<2$, while the absolute value of the right-hand side is at least $|-\sqrt{5}\cdot 3^{m}|\ge \sqrt{5}> 2$ for all $m>127$, which is a contradiction.

We apply Theorem 2 on the left-hand side of (13) with the data

$$\begin{aligned} t=3, ~~\gamma _1=\dfrac{\alpha ^{n-n_1}-1}{\sqrt{5}},~~ \gamma _2=\alpha , ~~\gamma _3=3, ~~ b_1=1, ~~b_2=n_1, ~~b_3=-m. \end{aligned}$$

The minimal polynomial of $\gamma _1$ divides

$$\begin{aligned} 5X^2-5F_{n-n_1}X-((-1)^{n-n_1}+1-L_{n-n_1}), \end{aligned}$$

where $\{L_{k}\}_{k\ge 0}$ is the Lucas companion sequence of the Fibonacci sequence given by $L_0=2, ~~L_1=1,~~L_{k+2}=2L_{k+1}+L_k$ for all $k\ge 0$, for which the Binet formula for its general term is given by

$$\begin{aligned} L_k=\alpha ^{k}+\beta ^{k} ~~\text { for all } k\ge 0. \end{aligned}$$

Thus, we obtain

14 $$\begin{aligned} h(\gamma _1)\le & {} \dfrac{1}{2}\left( \log 5+\log \left( \dfrac{\alpha ^{n-n_1}+1}{\sqrt{5}}\right) \right) ~<~\dfrac{1}{2}\log (2\sqrt{5}\alpha ^{n-n_1})\nonumber \\< & {} \dfrac{1}{2}(n-n_1+2)\log \alpha ~<~8.4\times 10^{11}(1+\log 2n). \end{aligned}$$

So, we can take $A_1:=1.67\times 10^{12}(1+\log 2n)$. Furthermore, as before, we take $A_2:=\log \alpha$ and $A_3:=2\log 3$. Finally, since $\max \{1, n_1, m\}\le 2n$, we can take $B:=2n$. Then, we get

$$\begin{aligned} \log |\Lambda _1|> & {} -1.4\cdot 30^6\cdot 3^{4.5}\cdot 2^2(1+\log 2)(1+\log 2n)(16.8\times 10^{11} (1+\log 2n))\\&\times (\log \alpha )(2\log 3). \end{aligned}$$

Then,

$$\begin{aligned} \log |\Lambda _1|>-1.72\times 10^{24}(1+\log 2n)^2. \end{aligned}$$

By comparing the above relation with (13), we get that

15 $$\begin{aligned} (m-m_1)\log 3 < 1.80\times 10^{24}(1+\log 2n)^2. \end{aligned}$$

**Case 2.**$$\min \{(n-n_1)\log \alpha , (m-m_1)\log 3\} = (m-m_1)\log 3$$.

In this case, we rewrite (6) as

$$\begin{aligned} \left| \dfrac{\alpha ^{n}}{\sqrt{5}}-(3^{m-m_1}-1)\cdot 3^{m_1}\right| =\left| \dfrac{\beta ^n+\alpha ^{n_1}-\beta ^{n_1}}{\sqrt{5}}\right| ~<~\dfrac{\alpha ^{n_1}+2}{\sqrt{5}}~<~\alpha ^{n_1}, \end{aligned}$$

which implies that

16 $$\begin{aligned} |(\sqrt{5}(3^{m-m_1}-1))^{-1}\alpha ^{n}3^{-m_1}-1|< & {} \dfrac{\alpha ^{n_1}}{3^{m}-3^{m_1}}~\le ~\dfrac{3\alpha ^{n_1}}{3^{m}}\nonumber \\< & {} 3\alpha ^{n_1-n+4}~<~\alpha ^{n_1-n+7}. \end{aligned}$$

We put

$$\begin{aligned} \Lambda _{2}=(\sqrt{5}(3^{m-m_1}-1))^{-1}\alpha ^{n}3^{-m_1}-1. \end{aligned}$$

Clearly, $\Lambda _2\ne 0$, for if $\Lambda _2 =0$, then $\alpha ^{2n}\in {\mathbb {Q}}$, which is a contradiction. We again apply Theorem 2 with the following data

$$\begin{aligned} t=3,~~ \gamma _{1}=\sqrt{5}(3^{m-m_1}-1), ~~\gamma _2=\alpha , ~~\gamma _3=\alpha , ~~b_1=-1, ~~b_2=n, ~~b_3= -m_1. \end{aligned}$$

The minimal polynomial of $\gamma _1$ is $X^2-5(3^{m-m_1}-1)^2$. Thus,

$$\begin{aligned} h(\gamma _{1})=\log (\sqrt{5}(3^{m-m_1}-1))<(m-m_1+1)\log 3 < 1.25\times 10^{12}(1+\log 2n). \end{aligned}$$

So, we can take $A_1:=2.5\times 10^{12}(1+\log 2n)$. Further, as in the previous applications, we take $A_2:=\log \alpha$ and $A_3:=2\log 3$. Finally, since $\max \{1, n, m_1\}\le 2n$, we can take $B:=2n$. Then, we get

$$\begin{aligned} \log |\Lambda _2|> & {} -1.4\cdot 30^6\cdot 3^{4.5}\cdot 2^2(1+\log 2)(1+\log 2n)(2.5\times 10^{12}\\&\times (1+\log 2n))(\log \alpha )(2\log 3). \end{aligned}$$

Thus

$$\begin{aligned} \log |A_2|>- 2.56\times 10^{24}(1+\log 2n)^2. \end{aligned}$$

Now, by comparing with (16), we get that

17 $$\begin{aligned} (n-n_1)\log \alpha < 2.58\times 10^{24}(1+\log 2n)^2. \end{aligned}$$

Therefore, in both Case 1 and Case 2, we have

18 $$\begin{aligned} \min \{(n-n_1)\log \alpha , (m-m_1)\log 3\}< & {} 1.24\times 10^{12}(1+\log 2n),\nonumber \\ \max \{(n-n_1)\log \alpha , (m-m_1)\log 3\}< & {} 2.58\times 10^{24}(1+\log 2n)^2. \end{aligned}$$

Finally, we rewrite the equation (6) as

$$\begin{aligned} \left| \dfrac{(\alpha ^{n-n_1}-1)}{\sqrt{5}}\alpha ^{n_1}-(3^{m-m_1}-1)\cdot 3^{m_1}\right| =\left| \dfrac{\beta ^{n}-\beta ^{n_1}}{\sqrt{5}}\right| ~<~|\beta |^{n_1}<1. \end{aligned}$$

Dividing through by $3^m-3^{m_1}$, we get

19 $$\begin{aligned} \left| \left( \dfrac{\alpha ^{n-n_1}-1}{\sqrt{5}(3^{m-m_1}-1)}\right) \alpha ^{n_1}3^{-m_1}-1\right|< & {} \dfrac{1}{(3^{m}-3^{m_1})}\le \dfrac{3}{ 3^{m}}\nonumber \\\le & {} 3\alpha ^{-(n-4)}~\le ~\alpha ^{7-n}, \end{aligned}$$

since $\alpha <3$ and $\alpha < \alpha ^{n_1}$. We again apply Theorem 2 on the left-hand side of (19) with the data

$$\begin{aligned} t=3, ~~\gamma _{1}=\dfrac{\alpha ^{n-n_1}-1}{\sqrt{5}(3^{m-m_1}-1)}, ~~\gamma _2=\alpha , ~~\gamma _3=3, ~~b_1=1, ~~b_2=n_1, ~~b_3=-m_1. \end{aligned}$$

Using the algebraic properties of the logarithmic height function, we get

$$\begin{aligned} h(\gamma _1)= & {} h\left( \dfrac{\alpha ^{n-n_1}-1}{\sqrt{5}(3^{m-m_1}-1)}\right) ~\le ~h\left( \dfrac{\alpha ^{n-n_1}-1}{\sqrt{5}}\right) +h(3^{m-m_1}-1)\\< & {} \dfrac{1}{2}(n-n_1+4)\log \alpha +(m-m_1)\log 3~<2.80\times 10^{24}(1+\log 2n)^2, \end{aligned}$$

where in the above inequalities, we used the argument from (14) as well as the bounds (18). Thus, we can take $A_1:=5.60\times 10^{24}(1+\log 2n)$, and again as before $A_2:=\log \alpha$ and $A_3:=2\log 3$. If we put

$$\begin{aligned} \Lambda _3 = \left( \dfrac{\alpha ^{n-n_1}-1}{\sqrt{5}(3^{m-m_1}-1)}\right) \alpha ^{n_1}3^{-m_1}-1, \end{aligned}$$

we need to show that $\Lambda _3\ne 0$. If not, $\Lambda _3=0$ leads to

$$\begin{aligned} \alpha ^{n}-\alpha ^{n_1}=\sqrt{5}(3^{m}-3^{m_1}). \end{aligned}$$

A contradiction is reached upon a conjugation in ${\mathbb {K}}$ and by taking absolute values on both sides. Thus, $\Lambda _3 \ne 0$. Applying Theorem 2 gives

$$\begin{aligned} \log |\Lambda _3|> & {} -1.4\cdot 30^6 \cdot 3^{4.5}\cdot 2^2 (1+\log 2)(1+\log 2n)(5.6\times 10^{24}(1+\log 2n)^2)\\&\times (\log \alpha )(2\log 3), \end{aligned}$$

a comparison with (19) gives

$$\begin{aligned} (n-4)< & {} 3\times 10^{36}(1+\log 2n)^3, \end{aligned}$$

or

20 $$\begin{aligned} 2n< & {} 6.2\times 10^{36}(1+\log 2n)^3. \end{aligned}$$

Now by applying Lemma 3 on (20) with the data $$ m=3 $$, $$ T=6.2\times 10^{36}$$, and $$ x=2n$$, leads to $$ n< 2\times 10^{40} $$.

### Reducing the bound for *n*

We need to reduce the above bound for *n* and to do so we make use of Lemma 2 several times. To begin, we return to (12) and put

$$\begin{aligned} \Gamma :=n\log \alpha -m\log 3 -\log (\sqrt{5}). \end{aligned}$$

For technical reasons, we assume that $\min \{n-n_1, m-m_1\}\ge 20$. We go back to the inequalities for $\Lambda$, $\Lambda _1$, and $\Lambda _2$. Since we assume that $\min \{n-n_1, m-m_1\}\ge 20$ we get $|e^{\Gamma }-1|=|\Lambda |< \frac{1}{4}$. Hence, $|\Lambda |<\frac{1}{2}$ and since the inequality $|y|<2|e^{y}-1|$ holds for all $y\in \left( -\frac{1}{2}, \frac{1}{2}\right)$, we get

$$\begin{aligned} |\Gamma |<2\max \{\alpha ^{n_1-n+5}, 3^{m_1-m+1}\}\le \max \{\alpha ^{n_1-n+8}, 3^{m_1-m+2}\}. \end{aligned}$$

Assume that $\Gamma >0$. We then have the inequality

$$\begin{aligned} 0<n\left( \dfrac{\log \alpha }{\log 3}\right) -m+\dfrac{\log (1/\sqrt{5})}{\log 3}< & {} \max \left\{ \dfrac{\alpha ^{8}}{(\log 3)\alpha ^{n-n_1}}, \dfrac{6}{(\log 3)3^{m-m_1}}\right\} .\\< & {} \max \{45\alpha ^{-(n-n_1)}, 8\cdot 3^{-(m-m_1)}\}. \end{aligned}$$

We apply Lemma 2 with the data

$$\begin{aligned} \tau = \dfrac{\log \alpha }{\log 3}, ~~~\mu = \dfrac{\log (1/\sqrt{5})}{\log 3}, ~~~(A,B)=(45, \alpha ) ~~\text { or } (8,3). \end{aligned}$$

Let $\tau = [a_{0}; a_{1}, a_{2}, \ldots ]=[0; 2, 3, 1, 1, 6, 1, 49, 1, 2, 2, 1, 1, 2, 1, 2, 2, 1, 10, 3, \ldots ]$ be the continued fraction of $\tau$. We choose $M:=2\times 10^{40}$ and consider the 91th convergent

$$\begin{aligned} \dfrac{p}{q}=\dfrac{p_{91}}{q_{91}}=\dfrac{487624200385184167130255744232737921512174859336581}{1113251817385764505972408650620147577750763395186265}. \end{aligned}$$

It satisfies $q=q_{91}>6M$. Furthermore, it yields $\varepsilon > 0.4892$ and, therefore, either

$$\begin{aligned} n-n_1\le \dfrac{\log (45q/\varepsilon )}{\log \alpha }< 254, ~~\text { or } ~~ m-m_1\le \dfrac{\log (8q/\varepsilon )}{\log 3}<110. \end{aligned}$$

In the case $\Gamma <0$, we consider the inequality

$$\begin{aligned} m\left( \dfrac{\log 3}{\log \alpha }\right) -n+\dfrac{\log (\sqrt{5})}{\log \alpha }< & {} \max \left\{ \dfrac{\alpha ^{8}}{\log \alpha }\alpha ^{-(n-n_1)}, ~\dfrac{8}{\log \alpha }\cdot 3^{-(m-m_1)}\right\} \\< & {} \max \{98\alpha ^{-(n-n_1)}, ~18\cdot 3^{-(m-m_1)}\}. \end{aligned}$$

We then apply Lemma 2 with the data

$$\begin{aligned} \tau = \dfrac{\log 3}{\log \alpha }, ~~ \mu = \dfrac{\log \sqrt{5}}{\log \alpha }, ~~ (A,B)=(98, \alpha ), ~~\text { or } ~ (18,3). \end{aligned}$$

Let $\tau = [a_0; a_1, a_2, \ldots ] = [2; 3, 1, 1, 6, 1, 49, 1, 2, 2, 1, 1, 2, 1, 2, 2, 1, 10, 3, 12, \ldots ]$ be the continued fraction of $\tau$. Again, we choose $M=2\times 10^{40}$, and in this case we consider the 101th convergent

$$\begin{aligned} \dfrac{p}{q}=\dfrac{p_{101}}{q_{101}}=\dfrac{106360048375891410642967692492903700137161881169662}{56228858848524361385900581302251812795713192394033}, \end{aligned}$$

which satisfies $q=q_{101}>6M$. Further, this yields $\varepsilon >0.125$ and, therefore, either

$$\begin{aligned} n-n_1\le \dfrac{\log (98q/\varepsilon )}{\log \alpha }<254 ~~~, \text { or }~~ m-m_1\le \dfrac{\log (18q/\varepsilon )}{\log 3}< 110. \end{aligned}$$

These bounds agree with the bounds obtained in the case $\Gamma >0$. As a conclusion, we have that either $n-n_1\le 253$ or $m-m_1 \le 109$ whenever $\Gamma \ne 0$.

Now, we distinguish between the cases $n-n_1\le 253$ and $m-m_1\le 109$. First, we assume that $n-n_1\le 253$. In this case, we consider the inequality for $\Lambda _1$, (13) and also assume that $m-m_1\le 20$. We put

$$\begin{aligned} \Gamma _1=n_1\log \alpha -m\log 3+\log \left( \dfrac{\alpha ^{n-n_1}}{\sqrt{5}}\right) . \end{aligned}$$

Then, the inequality (13) implies that

$$\begin{aligned} |\Gamma _1|<\dfrac{6}{3^{m-m_1}}. \end{aligned}$$

If we further assume that $\Gamma _1>0$, we then get

$$\begin{aligned} 0<n_{1}\left( \dfrac{\log \alpha }{\log 3}\right) -m+\dfrac{\log ((\alpha ^{n-n_1}-1)/\sqrt{5})}{\log 3}<\dfrac{6}{(\log 3)3^{m-m_1}}<\frac{6}{3^{m-m_1}}. \end{aligned}$$

Again we apply Lemma 2 with the same $\tau$ as in the case $\Gamma >0$. We use the 91th convergent $p/q=p_{91}/q_{91}$ of $\tau$ as before. But in this case, we choose $(A,B):=(8,3)$ and use

$$\begin{aligned} \mu _l=\dfrac{\log ((\alpha ^{l}-1)/\sqrt{5})}{\log 3}, \end{aligned}$$

instead of $\mu$ for each possible value of $l:=n-n_1 \in [1,2,\ldots , 253]$. We have problems at $l \in \{4,12\}$. We discard these values for now and we will treat them later. For the remaining values of *l*, we get $\varepsilon >0.0005$. Hence by Lemma 2, we get

$$\begin{aligned} m-m_1~<~\dfrac{\log (8q/0.0005)}{\log 3}~<~116. \end{aligned}$$

Thus, $n-n_1\le 253$ implies that $m-m_1\le 115$, unless $n-n_1\in \{4,12\}$. A similar conclusion is reached when $\Gamma _1<0$ with the same two exceptions for $n-n_1\in \{4,12\}$. The reason we have a problem at $l\in \{4, 12\}$ is because

$$\begin{aligned} \dfrac{\alpha ^{4}-1}{\sqrt{5}}=\alpha ^{2}, ~~~\text { and }~~\dfrac{\alpha ^{12}-1}{\sqrt{5}}=2^{3}\alpha ^{6}. \end{aligned}$$

So, $\Gamma _1=(n_1+2)\log \alpha -m\log 3$ , or $(n_1+6)\log \alpha -(m-3)\log 3$ when $l=4,12$, respectively. Thus we get that

$$\begin{aligned} \left| \tau - \dfrac{m}{n_1+2}\right|<\dfrac{6}{3^{m-m_1}(n_{1}+2)}, ~~\text { or } ~~ \left| \tau - \dfrac{m-3}{n_1+6}\right| <\dfrac{6}{3^{m-m_1}(n_{1}+6)}, \end{aligned}$$

respectively. We assume that $m-m_1>150$. Then, $3^{m-m_1}>8\times (4\times 10^{40})>8\times (n_1+6)$; therefore

$$\begin{aligned} \dfrac{6}{3^{m-m_1}(n_1+2)}<\dfrac{1}{3(n_1+2)^{2}}, ~~~\text { and } ~~ \dfrac{6}{3^{m-m_1}(n_1+6)}<\dfrac{1}{3(n_1+6)^{2}}. \end{aligned}$$

By Lemma 1, it follows that $m/(n_1+2)$ or $(m-3)/(n_1+6)$ are convergents of $\tau$, respectively. So, say one of $m/(n_1+2)$ or $(m-3)/(n_1+6)$ is of the form $p_k/q_k$ for some $k=0, 1, 2, \ldots , 92$. Here, we use that $q_{92}>4\times 10^{40}> n+1+6$. Then

$$\begin{aligned} \dfrac{1}{(a_{k}+2)q_k^2}<\left| \tau -\dfrac{p_k}{q_k}\right| . \end{aligned}$$

Since $\max \{a_{k}:k=0,1,2, \ldots ,92\}= 140$, we get

$$\begin{aligned} \dfrac{1}{142q_k^2}<\dfrac{6}{3^{m-m_1}q_k}~~~\text { and }~~ q_{k}~~ \text { divides one of }~~\{n_1+2, n_1+6\}. \end{aligned}$$

Thus, we get

$$\begin{aligned} 3^{m-m_1}\le 6\times 142(n_1+6)< 6\times 142\times 4\times 10^{40}, \end{aligned}$$

giving $$ m-m_1\le 92 $$.

Now let us turn to the case $m-m_1\le 109$ and we consider the inequality for $\Lambda _2$, (16). We put

$$\begin{aligned} \Gamma _{2}=n\log \alpha -m_1\log 3+\log (1/(\sqrt{5}(3^{m-m_1}-1))), \end{aligned}$$

and we also assume that $n-n_1\ge 20$. We then have

$$\begin{aligned} |\Gamma _{2}|<\dfrac{2\alpha ^{8}}{\alpha ^{n-n_1}}. \end{aligned}$$

We assume that $\Gamma _{2}$, then we get

$$\begin{aligned} 0~<~n\left( \dfrac{\log \alpha }{\log 3}\right) -m_1+\dfrac{\log (1/(\sqrt{5}(3^{m-m_1}-1))}{\log \alpha }~<~\dfrac{3\alpha ^{8}}{(\log 3)\alpha ^{n-n_1}}~<~\dfrac{130}{\alpha ^{n-n_1}}. \end{aligned}$$

We apply again Lemma 2 with the same $\tau , ~ q, ~ M, ~~ (A,B):=(130, \alpha )$ and

$$\begin{aligned} \mu _l=\dfrac{\log (1/(\sqrt{5}(3^{l}-1)))}{\log 3}~~\text { for } k=1, 2, \ldots , 109. \end{aligned}$$

We get $\varepsilon > 0.004$; therefore

$$\begin{aligned} n-n_1<\dfrac{\log (130q/\varepsilon )}{\log \alpha }<266. \end{aligned}$$

A similar conclusion is reached when $\Gamma _2<0$. To conclude, we first get that either $n-n_1\le 253$ or $m-m_1\le 109$. If $n-n_1\le 253$, then $m-m_1\le 115$, and if $m-m_1\le 109$ then $n-n_1\le 265$. Thus, we conclude that we always have $n-n_1\le 265$ and $m-m_1\le 115$.

Finally, we go to the inequality of $\Lambda _{3}$, (19). We put

$$\begin{aligned} \Gamma _3=n_1\log \alpha -m_1\log 3+\log \left( \dfrac{\alpha ^{n-n_1}-1}{\sqrt{5}(3^{m-m_1}-1)}\right) . \end{aligned}$$

Since $n\ge 300$, the inequality (19) implies that

$$\begin{aligned} |\Gamma _{3}|<\dfrac{3}{\alpha ^{n-4}}=\dfrac{3\alpha ^{4}}{\alpha ^{n}}. \end{aligned}$$

Assuming that $\Gamma _3>0$, then

$$\begin{aligned} 0<n_1\left( \dfrac{\log \alpha }{\log 3}\right) -m_1+\dfrac{\log ((\alpha ^{k}-1)/(\sqrt{5}(3^{l}-1))}{\log 3}<\dfrac{3\alpha ^{4}}{(\log 3)\alpha ^{n}}<\dfrac{20}{\alpha ^{n}}, \end{aligned}$$

where $(k,l):=(n-n_1, m-m_1)$. We again apply Lemma 2 with the same $\tau , ~~q, ~~M, ~~(A,B):=(20, \alpha )$ and

$$\begin{aligned} \mu _{k,l}=\dfrac{\log ((\alpha ^{k}-1)/(\sqrt{5}(3^{l}-1))}{\log 3}~~\text { for } ~~ 1\le k\le 265, ~~1\le l\le 115. \end{aligned}$$

As before, we have a problem at $(k,l):=(4,1), ~(12,1), ~ (8,2)$. The cases $(k,l):=(4,1), (12,1)$ were treated before in the case of $\Gamma _1$. The case $(k,l):=(8,2)$ arises because

$$\begin{aligned} \dfrac{\alpha ^{8}-1}{\sqrt{5}(3^{2}-1)}=\dfrac{3}{8}\alpha ^{4}. \end{aligned}$$

We, therefore, discard the cases $(k,l):=(4,1), ~(12,1), ~ (8,2)$ for some time. For the remaining cases, we get $\varepsilon > 0.0015$, so we obtain

$$\begin{aligned} n\le \dfrac{\log (20q/\varepsilon )}{\log \alpha }<264. \end{aligned}$$

A similar conclusion is reached when $\Gamma _{3}<0$. Hence, $n<300$. Now, we look at the cases $(k,l):=(4,1), ~(12,1), ~ (8,2)$. The cases $(k,l):=(4,1), ~(12,1)$ can be treated as before when we showed that $n-n_1 \le 263$ implies $m-m_1\le 115$. The case when $(k,l)=(8,2)$ can be dealt with in a similar way. Namely, it gives that

$$\begin{aligned} |(n_1+4)\tau - m_1|<\dfrac{20}{\alpha ^{n}}. \end{aligned}$$

Therefore

21 $$\begin{aligned} \left| \tau - \dfrac{m_1}{n_1+4}\right| <\dfrac{20}{(n_1+4)\alpha ^{n}}. \end{aligned}$$

Since $n\ge 300$, we have $\alpha ^{n}>2\times 20\times (4\times 10^{40})>40(n_1+4)$. This shows that the right-hand side of the above inequality (21) is at most $2/(n_1+4)^2$. By Lemma 1, we get that $m_1/(n_1+4)=p_k/q_k$ for some $k=1,2, \ldots , 92$. We then get by a similar argument as before that

$$\begin{aligned} \alpha ^{n}<20\times 142\times (4\times 10^{40}), \end{aligned}$$

which gives $n\le 211$. Therefore, the conclusion is that $n<300$ holds also in the case $(k,l)=(8,2)$. However, this contradicts our working assumption that $n>300$. This completes the proof of Theorem 1.
